# Automatic inference of indexing rules for MEDLINE

**DOI:** 10.1186/1471-2105-9-S11-S11

**Published:** 2008-11-19

**Authors:** Aurélie Névéol, Sonya E Shooshan, Vincent Claveau

**Affiliations:** 1 National Library of Medicine, 8600 Rockville Pike, Bethesda, MD 20894, USA; 2 IRISA – CNRS, Campus de Beaulieu, 35042 Rennes, France

## Abstract

**Background:**

Indexing is a crucial step in any information retrieval system. In MEDLINE, a widely used database of the biomedical literature, the indexing process involves the selection of Medical Subject Headings in order to describe the subject matter of articles. The need for automatic tools to assist MEDLINE indexers in this task is growing with the increasing number of publications being added to MEDLINE.

**Methods:**

In this paper, we describe the use and the customization of Inductive Logic Programming (ILP) to infer indexing rules that may be used to produce automatic indexing recommendations for MEDLINE indexers.

**Results:**

Our results show that this original ILP-based approach outperforms manual rules when they exist. In addition, the use of ILP rules also improves the overall performance of the Medical Text Indexer (MTI), a system producing automatic indexing recommendations for MEDLINE.

**Conclusion:**

We expect the sets of ILP rules obtained in this experiment to be integrated into MTI.

## Background

Information retrieval in either specialized or general databases calls for the *indexing *of documents. The index generated for each document is then used to match users' queries against the collection. Text documents are usually indexed using either *free-text indexing*, which consists of freely assigning sequences of words to the documents, or *controlled indexing*, which consists of assigning concepts available from a controlled list of terms; our work employs the latter.

In the biomedical domain, the Medical Subject Headings (MeSH^®^) thesaurus developed by the U.S. National Library of Medicine (NLM) is a widely used tool for indexing the literature. The MEDLINE database comprises more than 16 million biomedical articles. Each document referenced in MEDLINE is described by about a dozen keywords representing the subject matter of the article. These keywords are selected among the more than 24,000 MeSH main headings (e.g. *Aphasia*, *Patient Care*, *Hand*...). If appropriate, subheadings (or qualifiers) such as *surgery *or *pharmacology *chosen from a set of 83 may be attached to the main headings in order to refer to a more specific aspect of the concept. For each main heading, MeSH defines a subset of "allowable qualifiers", so that only certain pairs can be used as indexing terms. For example, the pairs *aphasia/metabolism *and *hand/surgery *are allowable, but *hand/metabolism *is not.

The steady increase of publications to be indexed in MEDLINE (about 2,500 per day) has led to the development of automatic tools such as the Medical Text Indexer (MTI) [1] intended to assist NLM indexers with indexing recommendations.

The complexity of the indexing task and the increasing amount of data to be processed make it necessary to improve existing tools. For automatic indexing in particular, the attachment of subheadings to the main heading recommendations produced automatically is a significant help to indexers. Recent work [2] in the context of the "Indexing 2015" project at NLM showed that the use of indexing rules applied to stand-alone main headings was a good method to produce pair recommendations. The use of qualifiers in the description of articles' subject matter usually follows implicit rules involving the main headings assigned to the paper. Sets of manual rules developed for a few subheadings show good performance in terms of precision but are often lacking in terms of recall. In addition, the development of new rules is a complex, time-consuming task.

The objective of the work presented here is to supplement the manual rules available with a set of automatically inferred rules to be used in MTI's Subheading Attachment module. We investigate a novel approach adapting Inductive Logic Programming (ILP) to the specific context of MEDLINE data, which requires efficient processing of large amounts of data. The rules that need to be obtained are similar to the following example: ***If ***a main heading from the "Anatomy" tree and a "Carboxylic Acids" term are recommended for indexing, ***then ***the pair " [Carboxylic Acids]/pharmacology" should also be recommended.

### Related work

The automatic indexing of the biomedical literature using MeSH indexing terms has been investigated by several researchers working on text in English as well as other European languages such as French, German and Portuguese. Indexing approaches include Natural Language Processing (NLP) [3], machine learning [4-6] or a combination of both [1,7]. However, due to the large number of indexing terms and even larger number of possible combinations most of these efforts, even the most recent [6], focus on stand-alone main headings and do not take subheadings into account. The experiments reported in this paper build on earlier work [2,3] addressing the issue of automatic indexing with MeSH main heading/subheading pairs.

To our knowledge, there has been no effort to automatically produce MeSH indexing rules. Related work [8] investigated the discovery of MeSH association rules to be used for query expansion in health information retrieval. However, few rules could be obtained because of the complexity of the problem description using usual machine learning techniques (see next section). The machine learning approach we chose to use, viz. ILP, is able to overcome these issues. ILP has been previously used for several machine-learning problems including NLP applications; for instance, it was used to infer extraction patterns [9], alignment rules [10], tagging and other tasks [11]. In these cases the expressiveness of ILP was found to be very useful; specifically, ILP was able to provide simple representations for relational problems and produce rules that can be easily interpreted. The ability of the method to accommodate complex representations usually translates into high computational complexity, which can result in prohibitive computation delays on large-scale problems. Therefore, special attention must be given to this issue as detailed in the next section.

## Methods

### Use of inductive logic programming

In this section, we briefly introduce the basic principles of ILP; a more comprehensive description can be found in [12]. After stressing the advantages of this method for MeSH indexing, we describe how it was adapted to accommodate our specific context.

#### Principles of inductive logic programming

ILP is a supervised machine learning technique used to infer rules that are expressed with logical clauses (Prolog clauses) based on a set of examples also represented using Prolog.

Let us consider a set of positive examples *E*^+^, a set of negative examples *E*^-^, and a background knowledge *B*. An ILP program aims at finding a set of rules *H *so that:

*B *∧ *H *∧ *E*^–^ |≠ □ and *B *∧ *H *|= *E*^+^, where □ represents false and |= represents a logical implication.

In practice, these two conditions can be relaxed so that, in fact, the set of rules explains (covers) most of the positive examples while rejecting most of the negative examples. This way, sparse or noisy data (frequently found in real-world applications) can be processed in order to obtain general rules that bear some exceptions.

**Figure 1:**
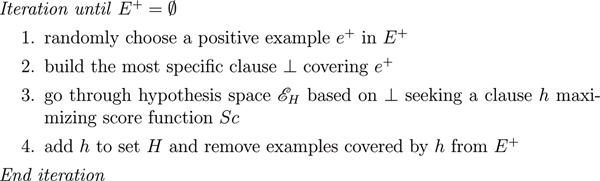
ALEPH Algorithm.

Most ILP algorithms deal with rule inference within a hypothesis space () as illustrated by Figure 1; the ILP tool that we used for our experiments, ALEPH [13], is based on this very algorithm. The method of exploring the hypothesis space  while constructing clauses is key for the inference phase (see last sub-section of this section).

#### Description of examples

In our context, ILP is used to infer rules describing the attachment of MeSH subheadings based on sample MEDLINE citations containing sets of MeSH indexing terms. For each subheading, we are expecting to find rules providing indications such as which main heading the subheading should be attached to, given the specific context the main heading appears in.

It may be argued that this problem can be addressed by a less complex method than ILP, such as association rules discovery [14]. However, methods for association rules discovery such as APRIORI would require describing each example as a tuple comprising the relevant main heading (that to which the subheading of interest may be attached) and the other main headings occurring in the citation. Basically, this would result in a tuple of dimension 24,000+ × 24,000+. Even less complex representations fail to account for the fact that a different number of main headings may be assigned to each citation and for the hierarchical structure of MeSH [8].

These issues, typical of propositional methods (i.e. methods describing problems as attribute/value tuples), are however naturally resolved by ILP. The expressiveness of Prolog allows for a simple description of relational problems. Let us consider the sample citation shown in Table 1. Occurrences of main headings to which the focus subheading is attached constitute positive examples. For example, let us take *pharmacology *as our focus subheading; according to the citation shown in Table 1 the following example *E*^+ ^will be constructed to indicate that *pharmacology *was attached to *Acrylamide*: qualif(mh16179550_1,"pharmacology"). Occurrences of main headings to which the focus subheading is not attached, although it is allowable for this main heading (such as *Astrocytes *in our sample citation), constitute negative examples. Contextual information for this occurrence (i.e. the other main headings assigned to the same citation) is accounted for in the background knowledge as shown in the table. The MeSH hierarchy is also included in the background knowledge in this way, with *W *representing a term:

**Table 1:** ILP representation of a MEDLINE citation

Excerpt of a sample citation	Excerpt of ILP representation in *B*
PMID – 16179550	in_article(pmid16179550, mh16179550_1).
MH – Acrylamide/*pharmacology	hierarchy(mh16179550_1,"Acrylamide").
MH – Animals	in_article(pmid16179550, mh16179550_2).
MH – Astrocytes/drug effects	hierarchy(mh16179550_2,"Animals").
MH – Histidine/physiology	in_article(pmid16179550, mh16179550_3).
MH – Amino Acid Transport	hierarchy(mh16179550_3,"Astrocytes").
Systems/*biosynthesis	in_article(pmid16179550, mh16179550_4).
...	...

hierarchy(W,"Neuroglia") :- hierarchy(W,"Astrocytes").

hierarchy(W,"Nervous System") :- hierarchy(W,"Neuroglia").

hierarchy(W,"Anatomy") :- hierarchy(W,"Nervous System").

...

This indicates that the main heading *Astrocytes *is a descendant of main heading *Neuroglia *in the MeSH hierarchy, which is, in turn, a descendant of *Nervous System*. MeSH hierarchical information described in this way can be exploited in the inference process. The construction of example clauses is fully automated. The rules that are then inferred use the various predicates described above. Below is a sample rule that could have been inferred from the example qualif(mh16179550_1,"pharmacology")., with *W *representing a term and *A *an article:

qualif(W_1_,"pharmacology") :- hierarchy(W_1_," Carboxylic Acids"), in_article(A, W_1_), in_article(A, W_2_), hierarchy(W_2_,"Anatomy"). In fact, it corresponds to the rule that we presented in the background section.

#### Optimizing the performance of the inference process

The consequence of the expressiveness of ILP is the complexity of rule inference. The hypothesis space  is usually very large, or even non-finite in some cases. Moreover, the computation of score *Sc *depends on the number of positive and negative examples covered (explained) by the hypothesis being tested, which implies checking every single example against the clause. This is the most cost-intensive aspect of ILP. It is necessary to handle it carefully in order to ensure reasonable computation times while producing relevant rules.

Luckily,  can be structured and inheritance properties can be taken into account while going through rules; this is commonly achieved using a *θ*-subsumption relation [15].

**Definition 1 (*θ*-subsumption) ***A clause C*_1 _*θ-subsumes (is more general than) a clause **if and only if *(*iff*) *a substitution θ so that C*_1_*θ *⊆ *C*_2 _*exists *(*considering clauses as literal sets*).

For example, the clause *C*_2 _= *p*(*a*, *b*) ← *r*(*b*, *a*) (i.e. *p*(*a*, *b*) :- *r*(*b*, *a*) in the standard Prolog format) is subsumed by *C*_1 _= *p*(*Y*_1_, *Y*_2_) ← *r*(*Y*_2_, *Y*_1_) since we have {*p*(*Y*_1_, *Y*_2_), ¬*r*(*Y*_2_, *Y*_1_)}*θ*_1 _⊆ {*p*(*a*, *b*), ¬*r*(*b*, *a*)} with *θ *= {*Y*_1_/*a*, *Y*_2_/*b*}.

Because of this hierarchical relationship between rules, the hypothesis space can be explored efficiently, for instance by moving from the most generic clause to the most specific one (⊥). From a clause such as *C*_1_, it is possible to generate more specific clauses covering only subsets of the examples covered by *C*_1_. This process contributes to speed up score computation for clauses that are more specific than *C*_1_. Similarly, it is possible to avoid generating a clause that will not cover enough examples as soon as it appears that an ancestor clause does not cover enough examples itself.

**Figure 2:**
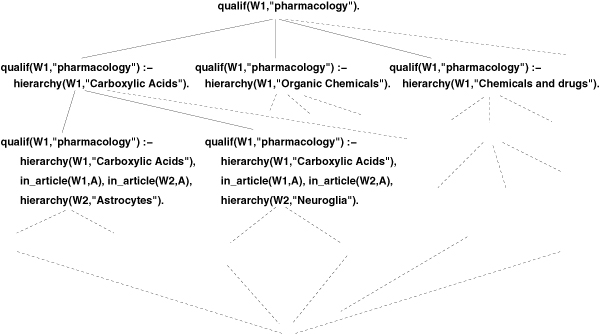
Excerpt of a search space structured by *θ*-subsumption.

However, *θ*-subsumption is not a perfect fit for our problem as it does not account for hierarchical relationships between MeSH main headings. Indeed, clause qualif(W_1_,"genetics") :- in _article(A, W_1_), in_article(A, W_2_), hierarchy(W_2_,"Frameshift Mutation") is not subsumed by qualif(W_1_,"genetics") :- in_article(A, W_1_), in_article(A, W_2_), hierarchy(W_2_,"Mutation") even though *Mutation *is an ancestor for *Frameshift Mutation *in the MeSH hierarchy. Figure 2 shows an excerpt of a lattice resulting from the organization of the search space with standard *θ*-subsumption. The very same problem appears since the clauses containing *Carboxylic Acids*, *Organic Chemicals *and *Chemical and Drugs*, are considered independantly although the main headings are related in the MeSH hierarchy. These hypotheses are thus evaluated independantly, and given the number of examples and possible hypotheses in the lattices, it results in very high computation times.

For this reason, we are introducing a new definition of subsumption based on work by Buntine [16] about *Generalized subsumption*:

**Definition 2 ***A clause C*_1 _*θ*_*hier*_*-subsumes a clause **with respect to background knowledge B iff there exist a substitution θ and a function f_D _such that f*_*D*_(*C*)*θ *⊆ *D*, *where f_D _is such that *∀*l *∈ *C*, *B*, *f*_*D*_(*l*) |= *l*, *with f*_*D*_({*l*_1_, *l*_2_,..., *l*_*m*_}) *meaning *{*f*_*D*_(*l*_1_), *f*_*D*_(*l*_2_),..., *f*_*D*_(*l*_*m*_)}.

**Figure 3:**
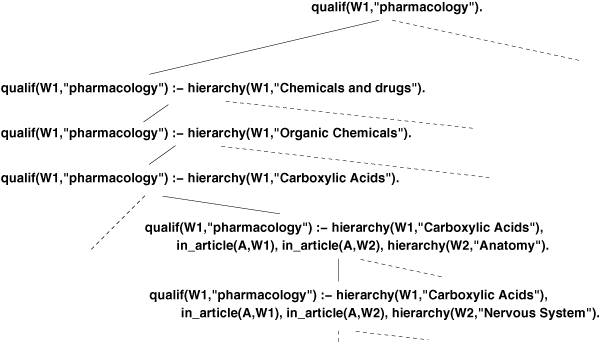
Excerpt of a search space structured by generalized subsumption.

Function *f*_*D *_accounts for information contained in the background knowledge, i.e. hierarchical relationships between MeSH terms in our case. This subsumption, implemented by modifying ALEPH, is consistent with the notion of coverage. It is easy to show that, given our background knowledge, this subsumption induces a partial order between clauses and thus also structures  as a lattice. Figure 3 shows the same excerpt as the one in Figure 2. Yet, with the use of the generalized subsumption, the lattice takes into account the relations described in the MeSH hierarchies. The lattice contains the same hypotheses as the one structured by standard *θ*-subsumption, but it is reorganized in a more hierarchical way. Thanks to the inheritance properties, it makes the computation of the score much more efficicent. This new subsumption is completely adapted to our context and allows us to efficiently infer rules from large sets of positive and negative examples (see "'Results"' section) while avoiding the computational pitfall of considering each of the 24,000 MeSH main headings independently. Note that, interestingly, this type of subsumption is in fact suitable for any rule inference problem involving structured knowledge as described in ontologies.

### Baseline method

Since association rules discovery methods are not completely adequate for our data, we have devised a simple baseline method for results comparisons. The baseline consists of random main heading/subheading attachments based on the statistical distribution of MeSH pairs in MEDLINE. The probability *P *of a main heading/subheading pair was computed as the number of occurrences of the pair in MEDLINE divided by the total number of occurrences of the corresponding main heading in MEDLINE, whether alone or attached to one or more subheadings. Table 2 presents the complete distribution for the main heading *Irritable Mood *and an excerpt of the distribution for *Lung*.

**Table 2:** MeSH subheading distribution in MEDLINE

*Irritable Mood*		*Lung *(excerpt)	
5 allowable qualifiers		26 allowable qualifiers	
**subheading**	***P***	**subheading**	***P***

no subheading	0.758	no subheading	0.047
classification	0.008	classification	0
drug effect	0.129	drug effect	0.082
ethics	0	metabolism	0.117
physiology	0.101	pathology	0.171
radiation effect	0.004	radiation effect	0.011

### Training and test corpora

ILP rules were induced using a training corpus comprising 100,000 citations randomly chosen from MEDLINE 2006. The statistical distribution of MeSH indexing terms used in the baseline method was derived from the 15,433,668 citations referenced in MEDLINE at the end of 2005 [17]. Finally, we also used a test corpus comprising 100,000 citations randomly chosen from MEDLINE 2006. Note that this corpus had no common citation with the training corpora mentioned above.

### Assessment of rules in a production indexing environment

In practice, as can be seen from the sample indexing rule in the introduction section, stand-alone main headings are used as trigger terms for applying the rules. For English, MTI[1], a tool developed at NLM, is able to retrieve stand-alone main heading indexing recommendations. On the test corpus, note that MTI retrieves about 45% of the main headings selected by NLM indexers for MEDLINE. For this reason, we anticipate that theoretical performance of ILP rules assessed directly on MEDLINE citations will be higher than the practical performance obtained by applying the rules to sets of terms retrieved by MTI, i.e. in a real production automatic indexing environment. The indexing rules inferred using ILP are assessed on the test corpus using MEDLINE indexing as a gold standard. ILP performance is then compared to that of manual rules produced by a domain expert and to the baseline described above.

For each method, (ILP, manual, baseline) only the pairs formed using a main heading recommended by MTI and also selected in MEDLINE are considered. The performance measures we used are precision, recall and balanced F-measure [18]. The significance of the differences in performance observed with the different methods was assessed with a pairwise T-test using an online analysis tool [19].

### Possible improvements on ILP rules

Preliminary experiments with producing ILP rules suggested that improvement could be achieved in two ways: (1) by filtering out rules that showed a comparatively low precision on the training corpus when applied to main headings retrieved by MTI; and (2) by having an indexing expert review the rules to improve their readability and reduce possible bias due to the training corpus. We report results of both improvement methods below.

## Results and discussion

Table 3 presents the theoretical performance for ILP rules (assessed on different MEDLINE citations than the ones used to infer the rules). In addition we also show the number of examples and the processing time on a desktop computer (Linux Intel Xeon 3 GHz).

**Table 3:** Performance of ILP rule inference on MEDLINE citations

**Subheading**	|***E***^+^|	|***E***^-^|	**Computing time**	**Precision (%)**	**Recall (%)**	**F-measure (%)**
*Administration & dosage*	5,300	40,000	75 mn	41.4	53.0	46.5
*Genetics*	5,700	30,500	51 mn	50.4	59.4	54.5
*Metabolism*	4,500	21,000	37 mn	42.4	60.2	49.7
*Pharmacology*	5,000	22,000	45 mn	48.8	53.9	51.2
*Physiology*	5,200	34,000	46 mn	41.4	41.5	41.5

Table 4 presents the performance on the test corpus for the ILP rules, compared to rules obtained manually (when available) and through the baseline method. We also show the performance obtained by ILP rules after filtering out rules that obtained a precision of less than 35% on the training corpus (ILP-filtered) and after review by a domain expert (ILP-reviewed). Here, the main headings triggering the application of rules were retrieved by MTI.

**Table 4:** Performance on the test corpus using MTI main heading recommendations

**Subheading**	**Method**	**No. rules**	**Precision (%)**	**Recall(%)**	**F-measure(%)**
*Administration & dosage*	ILP	166	38	**29**	**33**
	Manual	1	**54**	1	1
	ILP-filtered	95	45	25	32
	ILP-reviewed	124	37	**29**	**33**
	Baseline	-	26	9	13

*Genetics*	ILP	200	55	**39**	**46**
	Manual	226	**65**	28	39
	ILP-filtered	181	55	**39**	**46**
	ILP-reviewed	172	55	**39**	**46**
	Baseline	-	33	10	15

*Metabolism*	ILP	134	49	**38**	**43**
	Manual	61	**58**	20	30
	ILP-filtered	123	49	**38**	**43**
	ILP-reviewed	73	49	**38**	**43**
	Baseline	-	37	12	18

*Pharmacology*	ILP	217	47	**28**	**35**
	Manual	7	**67**	3	5
	ILP-filtered	183	48	**28**	**35**
	ILP-reviewed	74	47	**28**	**35**
	Baseline	-	28	12	17

*Physiology*	ILP	70	**46**	**24**	**32**
	Manual	0	-	-	-
	ILP-filtered	64	**46**	**24**	**32**
	ILP-reviewed	70	**46**	**24**	**32**
	Baseline	-	28	10	15

Table 5 presents the performance on the test corpus for MTI's subheading attachment module performing subheading attachment by combining four methods, including the use of post-processing rules. The other methods (dictionary method, MTI method and PubMed Related Citations) are detailed in [20]. Here, we compare the overall performance of the module using post-processing rules obtained manually, through ILP out-of-the-box and with a combination of manual and ILP rules, manual rules being used only when ILP rules were not available for a given subheading. Results for this last configuration are not shown because they were almost identical to those obtained with ILP. In table 4 and 5, the best performance for each subheading is bolded.

**Table 5:** Performance of MTI's subheading attachment module using Manual vs. ILP post-processing rules

**Subheading**	**PP rules**	**Precision (%)**	**Recall(%)**	**F-measure(%)**
*Administration & dosage*	ILP	**48**	**27**	**35**
	Manual	**48**	17	25

*Genetics*	ILP	55	**39**	**46**
	Manual	**56**	38	45

*Metabolism*	ILP	51	**47**	**49**
	Manual	**56**	33	42

*Pharmacology*	ILP	50	**38**	**43**
	Manual	**55**	24	33

*Physiology*	ILP	**45**	**32**	**37**
	Manual	44	23	30

*MeSH descriptors (83 Subheadings)*	ILP	49	**26**	**34**
	Manual	**50**	23	32

### General performance of ILP rules

As expected, the use of MTI to produce main heading recommendations used as triggers for the rules results in comparable precision but significantly lower recall compared to the theoretical assessment (*p *= 0.001). In spite of this, the performance obtained by ILP rules is statistically superior to the baseline and shows the best F-measure (*p *= 0.001). The precision obtained by the manual rules, when they exist, is higher (*p *= 0.007), but they produce a recall inferior to ILP (*p *= 0.007) and even to the baseline method, although in this case the difference was not statistically significant. It is important to note that our results compare favorably with the consistency between human indexers assigning MeSH pairs to articles referenced in MEDLINE, which was reported to be 33.8% [21].

#### ILP *vs*. manual rules

A detailed analysis of the rules obtained with ILP shows that, while some rules are easily understood by indexers: ***If ***a main heading from the "Neoplasms" subtree and a "Chromosomes" term are recommended for indexing, ***then ***the pair " [Chromosomes]/genetics" should also be recommended., others are not: ***If ***a main heading from the "Persons" subtree and a "Food and Beverages" term are recommended for indexing, ***then ***the pair " [Food and Beverages]/administration and dosage" should also be recommended.

This is due to some unexpected regularities which do not seem to be relevant but nonetheless achieved good results on the training data used to infer rules.

Furthermore, we noticed that while most rules typically contain a "trigger term" (e.g. *Persons *in our previous example) and a "target term" (e.g. *Food and Beverages *above), in some rules the target term can also serve as the trigger term. That is the case for example in ***If ***a main heading from the "Chemicals and Drugs" tree and an "Enzymes and Coenzymes" term are recommended for indexing, ***then ***the pair " [Enzymes and Coenzymes]/metabolism" should also be recommended., where *Enzymes and Coenzymes *is a descendant of *Chemicals and Drugs*. These rules may be explained by looking at the distribution of subheadings for the terms involved. For example, for *DNA Ligases*, a descendant of *Enzymes and Coenzymes *with 32 allowable qualifiers, *P*(*metabolism*) = 0.45. Some changes in the ILP inferring process are planned in order to prevent the production of such rules.

#### Rule filtering *vs*. manual review

The filtering process had the biggest impact on *administration and dosage *where 71 rules out of 166 had a precision lower than 35% on the training corpus. As a result, the precision on the test corpus improved by 7 points with only a 3 point decrease in recall and no impact on the F-measure. For the other subheadings studied here, filtering had little impact but generally tended to improve precision while F-measure stayed the same, which was our goal. On the other hand, the manual review of the rules seemed to degrade the performance obtained with the original ILP. Furthermore, the manual review of the rules is more costly than filtering, which can be done automatically. However, the differences in performance between ILP, ILP-reviewed and ILP-filtered were not statistically significant.

### Integrating ILP rules into a subheading attachment feature for MTI

#### Considerations for rule integration

One issue with the integration of ILP rules into MTI's subheading attachment feature is the fusion of ILP rule sets with the manual rule sets when they exist. Manual rules yield very good precision; therefore we may want ILP rules to *supplement *the manual sets instead of replacing them. However, while ILP rules and manual rules are generally distinct (only 6 rules were strictly identical), some cases of partial overlap were found. For example, the manual rule: ***If ***a main heading from the "Drug Resistance" subtree and a "Chemical Actions and Uses" term are recommended for indexing, ***then ***the pair "[Chemical Actions and Uses]/pharmacology" should also be recommended. and the ILP rule: ***If ***a main heading from the "Chemical and Pharmacologic Phenomena" subtree and a "Anti-Infective Agents" term are recommended for indexing, ***then ***the pair "[Anti-Infective Agents]/pharmacology" should also be recommended. are partially overlapping as *Anti-Infective Agents *is a descendant of *Chemical Actions and Uses *while *Chemical and Pharmacologic Phenomena *is an ancestor of *Drug Resistance*. We need to decide how these cases should be handled (i.e., keep both rules in final set or select manual or ILP rule).

#### Performance of rules in an indexing system

As shown in Table 5, the performance observed on the rules alone have a similar impact on the overall performance of MTI's subheading attachment module. Recall is higher (*p *= 0.008) when ILP rules are used (*vs. *manual rules), while there is no statistical difference in precision. We also compared these results to the performance obtained when all rules (manual and ILP) are applied, and there was no statistical difference with the use of ILP rules only. Therefore, in the final system, for each subheading, it seems sound to use whichever set of rules (Manual or ILP) yields the best results as this strategy will ensure the best performance is obtained with minimal integration effort.

## Conclusion

We have shown that it is possible to exhibit and exploit the regularities in the descriptions of biomedical articles indexed for MEDLINE to make automatic recommendations for the attachment of subheadings to main headings in new articles to be processed. Subheading attachment has been performed manually until now and the automation of this complex and time-consuming task has not received much attention in the past. However, the use of ILP provided us with a suitable representation thanks to the use of a form of subsumption specifically tailored to the MEDLINE context. This particular feature enabled the processing of large amounts of data to produce relevant indexing rules with reasonable computation times. This approach may also be applied to rule discovery in other large-scale datasets involving structured data (e.g., ontologies).

In the ILP experiments presented in this paper, the score *Sc *used to choose the best hypotheses in the search spaces was such that it gave the same weight to precision and recall. Different score functions are currently under study in order to favor more precise rules (yet keeping good F-measure); thus, it would make the post-filtering step superfluous. From a practical point of view, further work will be necessary in order to obtain rules for all 83 MeSH subheadings. In any case, we anticipate that the subheading attachment rule sets produced will be integrated into MTI's subheading attachment module.

## Competing interests

The authors declare that they have no competing interests.

## Authors' contributions

AN designed the study, prepared the data, performed the evaluation of the rules and of the subheading attachment module including the rules, and prepared the first draft of this manuscript. SES produced the manual rules, analyzed and reviewed the ILP rules and resulting indexing recommendations. VC implemented the ILP-based inference of the rules using the generalized subsumption operator, generated the rules and provided theoretical results on the performances and efficiency of the inference process. All authors read and approved the final manuscript.
